# Unprecedented levels of ultrafine particles, major sources, and the hydrological cycle

**DOI:** 10.1038/s41598-022-11500-5

**Published:** 2022-05-06

**Authors:** Wolfgang Junkermann, Jorg Hacker

**Affiliations:** 1 Karlsruhe Institute of Technology, KIT, IMK-IFU, Kreuzeckbahnstr. 19, 82467 Garmisch-Partenkirchen, Germany; 2 Airborne Research Australia, Parafield Airport, Hangar 60, Dakota Drive, 5106, Salisbury South, South Australia Australia; 3 College of Science and Engineering, Flinders University, Adelaide, South Australia Australia

**Keywords:** Climate sciences, Environmental sciences

## Abstract

Ultrafine particles (UFP) acting as cloud condensation nuclei (CCN) are the driving force behind changing rainfall patterns. Recently observed weather extremes like floods and drought might be due to changing anthropogenic UFP emissions. However, the sources and budgets of anthropogenic primary and secondary particles are not well known. Based on airborne measurements we identified as a major contribution modern fossil fuel flue gas cleaning techniques to cause a doubling of global primary UFP number emissions. The subsequent enhancement of CCN numbers has several side effects. It’s changing the size of the cloud droplets and delays raindrop formation, suppressing certain types of rainfall and increasing the residence time of water vapour in the atmosphere. This additional latent energy reservoir is directly available for invigoration of rainfall extremes. Additionally it’s a further contribution to the column density of water vapour as a greenhouse gas and important for the infrared radiation budget. The localized but ubiquitous fossil fuel related UFP emissions and their role in the hydrological cycle, may thus contribute to regional or continental climate trends, such as increasing drought and flooding, observed within recent decades.

## Introduction

Many of the extreme weather incidents that have been observed increasingly within recent decades are linked to changes of the hydrological cycle. Among these are modifications of the spatio-temporal patterns of rainfall, either missing rainfall, i.e. a reduction of the annual amount, or a shift to longer drought periods, or too much rainfall at once, an increase in frequency and intensity of torrential rain events and flooding^1^. Climate models link such observations to increasing carbon dioxide and correspondingly higher water vapour capacity of a warming atmosphere with ~ 7% more water per ~ 1 °C of global warming. The additional water vapour acts as a reservoir of latent heat and is thus able to invigorate convective processes which go along with a reconversion of water vapour to liquid water. However, as CO_2_ is spatially rather uniformly distributed due to its long lifetime, it is difficult to explain the extremely variable and uneven changes in the distribution and occurrence of rainfall with a carbon dioxide and corresponding water vapour increase alone. Instead, to explain such trends, additional processes that change residence time or the distribution of the shorter living climate relevant components water vapour and clouds need to be considered. The rather uneven and spatially poorly known distribution and the variation of the number concentration of aerosol particles acting as cloud condensation nuclei (CCN) seems to be a good candidate.

Compared to early reports from the 70ies, in regions prone to rainfall trends, enhanced number concentrations of aerosol particles in a size range invisible for the human eye, ultrafine particles (UFP, < 100 nm), were observed^2–5^. These UFPs are either precursors or direct cloud condensation nuclei (CCN), which reduce certain types of rainfall^6,7^ and increase the residence time of water vapour and liquid water in the atmosphere^1^.

How many aerosols of the corresponding sizes are contributing to CCN abundance depends on the individual sources, size, and chemistry^6^. Sources are either primary emission or secondary gas to particle conversion (GPC). Concerning the formation in the atmosphere by GPC, a variety of pathways is discussed in the literature in connection with short term ‘new particle formation’ (NPF) events, i.e. processes leading to the rapid change of nanoparticles < 10 nm in the planetary boundary layer (PBL), see for example^8^ and references cited therein. Sulphur and ammonia compounds and low volatility organics are amongst the key chemicals identified in such atmospheric continental nanoparticles. In maritime particle and CCN production the emission of dimethyl-sulphide dominates^9^. Yet, despite several decades of research on atmospheric nucleation, a quantitative estimate of the contribution of primary and secondary processes to the global particle number or the CCN budget is still outstanding. Recent aerosol models still underestimate the number concentrations compared to the global network of field sites^10^, indicating that either our chemical understanding of gas to particle conversion^11,12^ is incomplete or our estimates of primary particle emissions in that size range are incorrect.

Due to the short and size-dependent lifetime of UFPs, the spatial distribution of these particles (nucleation and Aitken mode) is extremely inhomogeneous^13,14^ and number concentrations often increase with altitude^5,15–18^. While this distribution makes quantitatively estimating ‘typical’ concentration levels rather difficult, the frequent plume-like shape of the highest concentration regions^19^ allow for identification and characterization of major sources. As the occurrence of such ’hotspots’ is independent of land use and vegetation patterns, sources of nanoparticles such as observed in NPF events may not be directly associated with biogenic emissions by vegetation, but rather with localized emissions from upwind anthropogenic sources. Nevertheless, VOCs likely contribute to particle aging and growth. As UFPs are invisible, the only currently available tool to estimate the amount of potential CCN are in-situ airborne measurements.

Correspondingly, we made detailed aircraft studies with small environmental research aircraft. Aerosol ‘hotspots’ with number concentrations above 20,000 cm^−3^ and up to^20,21^ 180,000 cm^−3^ proved to be clearly attributable to single sources, emitting UFPs into elevated layers of the daytime PBL or the residual layer (RL) during nighttime. We were able to follow such emissions over considerable distances up to > 1000 km, and even to quantify their source strength^4,14,20,21^. Details of airborne campaigns, platforms, instrumentation, data analysis and detailed results are included in the supplement.

Our flight campaigns covered areas from Northern Europe, the boreal forest in Finland, rural and industrialized Germany, Ireland, Great Britain, Southern France, and Northern Italy to the very south of Europe, the islands of Corsica, Malta and Lampedusa, as well as outside Europe, Mexico, Kenya, China (Inner Mongolia) and large sections of Australia from Western Australia to Northern Queensland. As such, the dataset is arguably the by far most comprehensive UFP-size distribution observational study ever conducted (see supplementary Table S2).

Study locations worldwide were selected based on optimum observation conditions for selected atmospheric climate-relevant processes, for example highly variable aerosol loads affecting radiation transfer, Saharan dust, biomass burning or aerosol industrial pollution (Italy-Lampedusa island and Po Valley), trace gas exchange and flux over different land use areas (China-Inner Mongolia, Germany), investigation of nucleation mode aerosol events (Finland, Ireland, Italy^44^), or characterization of aerosol, air chemistry and pollution levels (Southern France^29^, Malta and Corsica^5^). The number concentration of aerosols and the ratio of fine and ultrafine particles, together with a complete set of meteorological parameters, were used as key parameters for air mass and vertical stratification characterization. Some of the major results on UFP were found by chance, often even during ferry flights from home airfield to the main observation area, and were not initially addressed within the aims of the individual projects.

In Australia, the selection of flight regions and patterns was based on climate trend maps provided by the Australian Bureau of Meteorology (http://www.bom.gov.au/climate/). Initial results from Western Australia, where rainfall has been declining since about 1970 despite increasing water vapour concentrations^3,23^, indicated a significantly higher particle number load over the drought affected southwestern tip of the continent compared to the natural vegetation further inland (see also^24,25^). In Queensland we followed up on the hypothesis, that the observed rainfall decline was likely due to enhanced particle number concentrations^2^. Model investigations indicate that a marked change in particle emissions from anthropogenic sources at one time related better to regional scale rainfall decline than to a slow climate change related response^25,26^.

## Results and discussion

**Figure 1:**
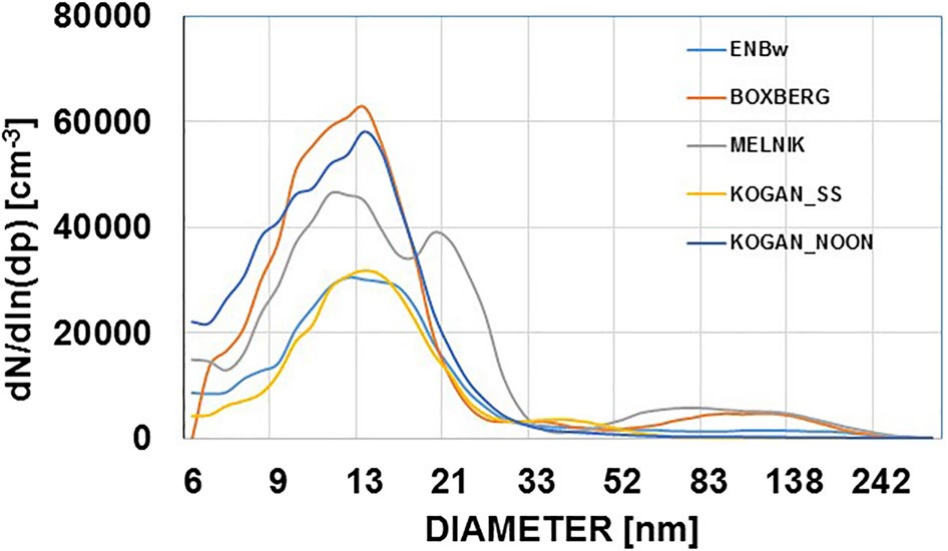
Aerosol size distributions measured ~ 1 h downwind of coal fired power stations (lignite and hard coal). Light blue: ENBw, Karlsruhe, July 2007; Orange: Boxberg, Eastern Germany, June 2014, both within the well mixed PBL at ~ 600 to 800 m AGL., Grey: Melnik, CZ observed early morning over Eastern Germany after 130 km nocturnal transport in the lower free troposphere > 600 m AGL, June 10, 2014, above a strong temperature inversion at ~ 550 m AGL. Yellow: Kogan Creek, Queensland, Australia at sunset (SS) and blue (noon, /2) the next day 25./26.8.2012, all within the mixed planetary boundary layer ~ 300 to 500 m AGL All size distributions shown are averages of a minimum of five subsequent DMA-scans within a plume, and quality controlled with concurrent fast condensation counter measurements.

In all our studies, even in typically rural or pristine environments such as the Inner Mongolian grasslands or the Australian outback, we found surprisingly high, but rather localised, concentrations of nanometer-sized particles with a pronounced nucleation mode radius between 3 and 10 nm (Fig. 1). Minimum PBL concentrations over the Australian outback were ~ 800 cm^−3^, slightly higher than the Bigg and Turvey^24^ estimate of 680 cm^−3^ while maxima over the outback reached well above 10^5^ cm^−3^. Similarly, over the grasslands of Inner Mongolia concentrations varied from between ~ 800 to 1000 cm^−3^ to 80,000 cm^−3^ within a few km^4^. Over continental areas in Europe, maximum concentrations reached > 90,000 cm^−3^, with background values between 1500 and 5000 cm^−3^. Even in places considered remote maritime, e.g. the islands of Lampedusa and Corsica, or the Great Barrier Reef on the east coast of Australia, low values of ~ 800 cm^−3^ as reported for the Mediterranean in 1972^27^ or for the southern ocean of^17^ 300–600 cm^−3^, have been found only occasionally in our campaigns since 1998. More typical lower PBL background values over the past two decades were^5,14,28,29^ ~ 1200 to 4000 cm^−3^. Over the islands of Malta and Gozo, located downwind of a major shipping route through the Mediterranean, the highest values at times reached > 150,000 cm^−3^. Concurrent continuous measurements on a rooftop on the island of Malta for the month of June 2013 went up to 65,000 cm^−3^ with a clear diurnal pattern reflecting the local-scale thermal island advection^5^, very similar to long term monitoring results from the Gozo Global Atmospheric Watch (GAW) station. Interestingly, the fine particulate mass or number concentration shows far lower variability than the UFP and is not correlated to any clear UFP plumes, although it occasionally can be used to characterize stratiform layers. Hence, the comparison of fine particles and UFP supports the hypothesis that, especially within the ultrafine fraction, major sources dominate the 3D distribution, while sources of fine particulate matter are more diffuse. We did not observe any further growth from the Aitken range into the fine fraction but also cannot exclude it.

The major UFP sources identified and quantified during these campaigns were fossil fuel burning emitters, power stations, refineries, smelters and ships. In situ meteorological observations combined with HYSPLIT^30^ analyses allow to investigate transport and meteorology over the time window relevant for the lifetime of nanometer-sized particles: lifetime and aging are different during convective daytime and during horizontal transport in a nocturnal stable residual layer.

A typical state of the art ‘clean’ coal fired power station with ~ 600 MW installed power emits about 1–2* 10^18^ s^−1^ or ~ 5* 10^25^ a^−1^ nanoparticles^4,14,20^. Emission rates and size distributions were found to be independent of the time of day with sunset values equal to midday values^20^, Fig. 1. Such particle sources emit primary particles rather than in-plume atmospheric GPC generated ones. Following^11^ or^22^, GPC related growth would not be fast enough to explain the observed size modes within the short time (~ 1 h) after release^12^. Size distributions found early morning in air masses transported overnight do not differ from emissions during the previous evening (Fig. 1). Also, during nocturnal transport in the residual layer, decoupled from the PBL, aerosol aging is not necessarily leading to growth^14,21^. Our observations of primary particle emissions point instead to an internal formation process, for example via ammonia addition for NO_*x*_ reduction^31,32^. This can explain the increasing CCN concentrations observed since the introduction of flue gas cleaning installations subsequent to clean air legislative initiatives, despite currently declining atmospheric sulphur dioxide concentrations^33^. A mixture of SO_*x*_, NO_*x*_ and ammonia at elevated temperatures would be favorable for production of both major components (NH_4_)_2_SO_4_ and NH_4_NO_3_^31,34^ and a good chemical base composition for CCN activity (6). In this case an agricultural contribution of ammonia^34–36^, is not required for the nucleation process, and it would also not result in the patchy UFP spatial distributions observed. However, the remaining ammonia after suppression of NO_*x*_, i.e. the unreacted ammonia slip (~ 1 to 2% of the amount applied and the emission for the inventories) is still a significant component of total NH_3_ emissions^37^. Similarly to biogenic emissions, it contributes to further growth of the UFPs to fine particulate matter (PM2.5)^36^.

Comparing our results to the emission scenario for the year 2000^38^, that is frequently used in current global climate models^39^, the most obvious difference is the smaller mode radius of the power generation related particles (5–10 nm instead of 500 nm), increasing the number emission by orders of magnitude (see also Fig. 4). With the resulting ~ 1.3 * 10^30^ particles a^−1^, only from fossil fuel power generation, the total global anthropogenic particle emission calculated from mass and mode radius would be approximately doubled. This number emission estimate, which critically depends on the selection of the radius, does not take into account any reduction of particulate mass (PM) emission since 2000 following PM control legislations. Yet even assuming 50% less mass it is roughly in agreement with an extrapolation of the average measured emission from several clean fossil fuel power stations to the > 10,000 such emitters worldwide (www.endcoal.com) (~ 5 * 10^29^ a^−1^). Despite already being far higher than the recent global continental particle number emission scenario (~ 3 * 10^27^ a^−1^) from power generation^40^, these numbers still represent only a lower estimate as other units burning fossil fuel, such as oil power stations, refineries, and smelters are not taken into account.

**Figure 2:**
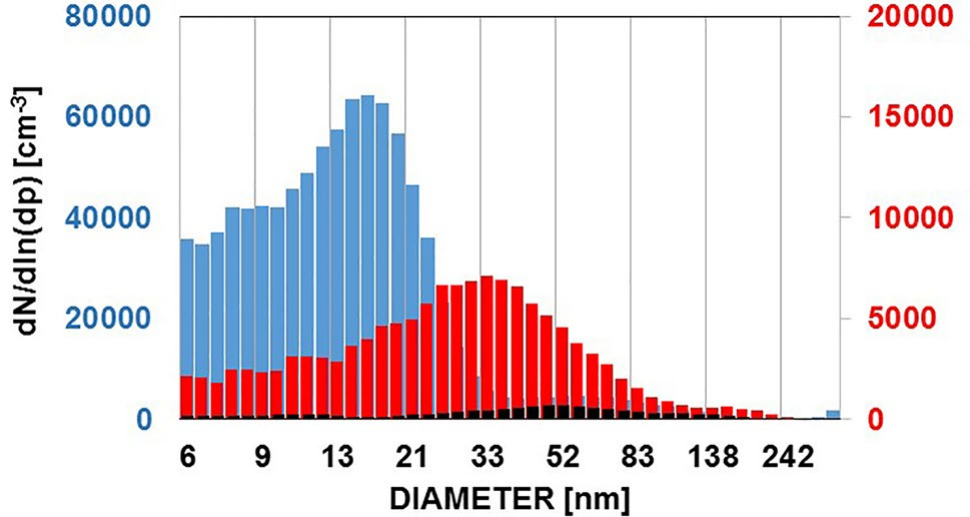
Size distributions measured downwind of shipping lines. Blue: over the islands of Malta/Gozo, June 14, 2013, max concentrations up to 150,000 cm^−3^, 2 h from the main shipping route. Horizontal transects over both islands below 600 m ASL, vertical profiles (see supplement) over Gozo between 50 m AGL and 3000 m ASL. Red (right scale): downwind (40 min) of a ‘clean’ Hybrid ferry in the Baltic Sea under nearly total (7/8) cloud cover and a maritime boundary layer of 500 m depth (HYSPLIT). Black (right scale): free troposphere above Malta and Gozo above 800 m ASL up to 3000 m ASL.

Besides continental sources, we investigated shipping emissions over the Mediterranean in an area without major pollution control and over the Baltic Sea in an emission control area (SECA). Ships with state of the art flue gas cleaning were found to be similarly effective UFP generators as ‘dirty’ ships, emitting particle sizes of about 15–20 nm mode radius (Fig. 2). Both, continental ‘clean’ and maritime ‘clean’ sources, agree within ~ 30% with a source strength of ~ 3 * 10^15^ particles^41^ MW^−1^ s^−1^.

For ships, the year 2000 ‘present day’ scenario of^38^ assumes a 0.5 µm mode radius which is similar to that of continental power generation. Hobbs^41^ and Petzold^42^ measured ~ 40 nm from bulk carriers, and our recently observed mode radii are even smaller, with 20 nm in the SECA area, and 8–10 nm over the Mediterranean (see Fig. 2). Considering these radii together with the doubling of ship traffic since 2000^43^, the resulting annual number emission for 2020 is similar to continental coal fired power stations (~ 3 * 10^29^ a^−1^).

The order of magnitude from these estimates agrees with our experimental investigations of the emissions from a line source between Malta and Sicily based on the relation between transported tonnage, ship size and engine power requirement for typical container ships^41^. The horizontal particle flux over the islands Malta and Gozo in June 2013 was estimated from vertical profiles of number concentrations, marine boundary layer thickness and horizontal perpendicular wind speed measured in situ^5^ (ship data were taken from AIS, www.marinetraffic.com, confirmation of wind speed and air mass history from HYSPLIT). An example of vertical profiles of UFP, fine particles and dewpoint is given in the supplement.

We can compare our results of primary emissions to current estimates of GPC in the atmosphere from nanoparticle observations, NPF events, a source considered as a significant planetary boundary layer contribution to CCN^34,44,45,46^. Using published numbers from long-term observations of NPF^45^, for example, the particle production over the Finnish boreal forest (~ 29,000 km^2^, PBL ~ 1000 m)^47^ equals about the magnitude of the primary emission of one or two mid-size fossil fuel power stations. Similar values result from box model considerations based on our airborne experiments at Hyytiälä, Finland^4,47^. Higher formation rates observed in China (North China Plain and Beijing)^46^ with ~ 13 particles cm^-3^ s^−1^, and peak values well above 200 cm^-3^ s^−1^ for 5 nm particles result in a similar equivalent production of about two mid-size power stations for the capital area of Beijing, 4567 km^2^. However, these estimates do not take into account that a major fraction of ground-based field site observed nanoparticles is due to advection from elevated, residual, layers aloft (> 42%)^18^, or from an undefined horizontal advection^44,47^, and should thus be taken as an upper limit only.

**Figure 3:**
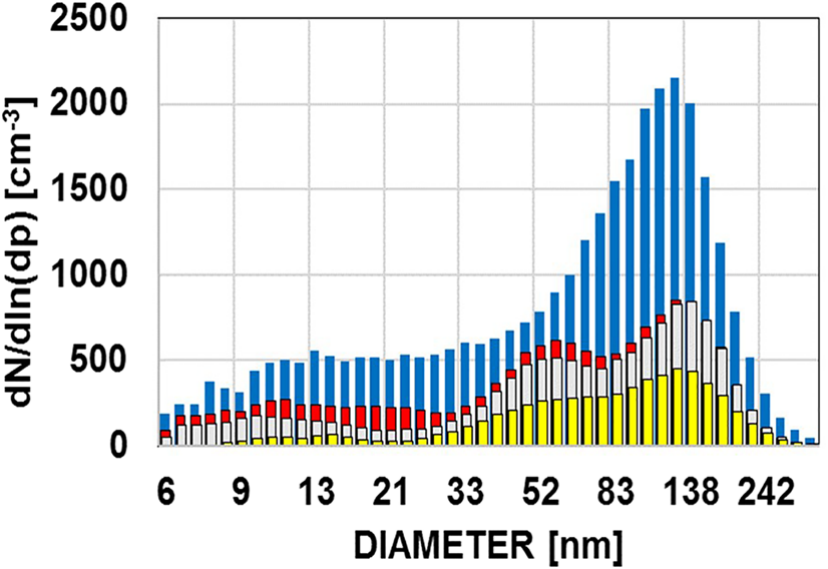
Size distributions measured under ‘remote’ coastal or marine conditions, averages of ~ 20 size distributions. Blue: 2–3 km above MSL over Corsica, aged particles ~ 4 to 5000 cm^−3^. Over a more than two week campaign, more than 80% of all particles were classified as cloud condensation nuclei (CCN). Grey: over the forested escarpment, continental coastline north of Townsville QLD, Australia, August 2012 (~ 1600 cm^−3^) all ~ 300 to 500 m above AGL. Red: 600 m ASL over the Great Barrier Reef, Australia, August 2012, north of Townsville (~ 2000 cm^−3^) ship pollution signature at 50–60 nm. Yellow: over the flat terrain at Cape Flattery, Queensland, Australia, (~ 1200 cm^−3^), see also^21^.

It is well known that new particles are formed by GPC within nucleation precursor rich air, e.g. aging plumes^11,12^. Signatures of such new particle formation with particles appearing in the nucleation mode could be observed occasionally under high UV radiation conditions over the Australian Outback and over the Mediterranean^20,21^ (Figs. 2, 3). This process, however, is limited to at least moderate pollution, fair weather, low cloud amount, and a limited number of hours during days with sufficient UV-radiation^11,22,48^.

## Summary and conclusions

Airborne measurements of ultrafine particles in nucleation and Aitken modes allowed an identification and quantification of major sources of ultrafine particles serving as CCN or their precursors. Fossil fuel derived ultrafine particles from continental sources and from shipping today equate to approximately twice the annual anthropogenic particle number emission when compared to the ‘present day’ year 2000 estimates^38,49^ used for current climate models, and are orders of magnitude larger than the recent estimate of the GAINS emission database gridded continental nanoparticle emissions^40^. The emissions are spatially highly uneven, but from ubiquitous distributed sources. This change in anthropogenic particle emissions is most likely a byproduct of the current state-of-the-art cleaning technologies for fossil fuel burning flue gas installed worldwide within the last two decades as consequence of clean air legislation since ~ 1990. The subsequent large increase of potential CCN, with highly variable regional enhancements, may have a severe impact on the hydrological cycle^4,50–53^. Today’s condensation nuclei (CN) and CCN (> ~ 60 nm) along the coastline of Queensland have increased by more than a factor of four compared to 1975^20,24^, average CN over Western Australia and over the central Mediterranean by more than an order of magnitude^5,27,54^.

Emitting the particles into the mid-levels of the PBL during daytime or into the RL above the PBL at night^21^ is favouring aerosol cloud interaction (ACI). The impact on cloud droplet size distributions is clearly manifest in satellite imagery as maritime or ‘continental’ ship tracks^21,50,51^. In addition to cloud brightening, smaller droplets have a series of hydrological consequences, physically plausible, but hardly accessible to experimental investigation. They not only delay the raindrop production and suppress certain types of regional rainfall^51,52,55^, thus increasing the atmospheric H_2_O residence time, but also lead to a lower liquid water content compared to clean clouds via more rapid evaporation of the smaller droplets^1,4,56^. Further transport of water vapour, and thus latent heat, from the boundary layer into the lower free troposphere between cumulus clouds may be an additional process enhancing the latent energy reservoir^57^, the regional water vapour abundance above the PBL, and the H_2_O column density^58,59^. In addition to an invigoration of convective torrential rains^60^ this also directly affects the infrared radiation balance^61^ through increased downward terrestrial radiation.

**Figure 4:**
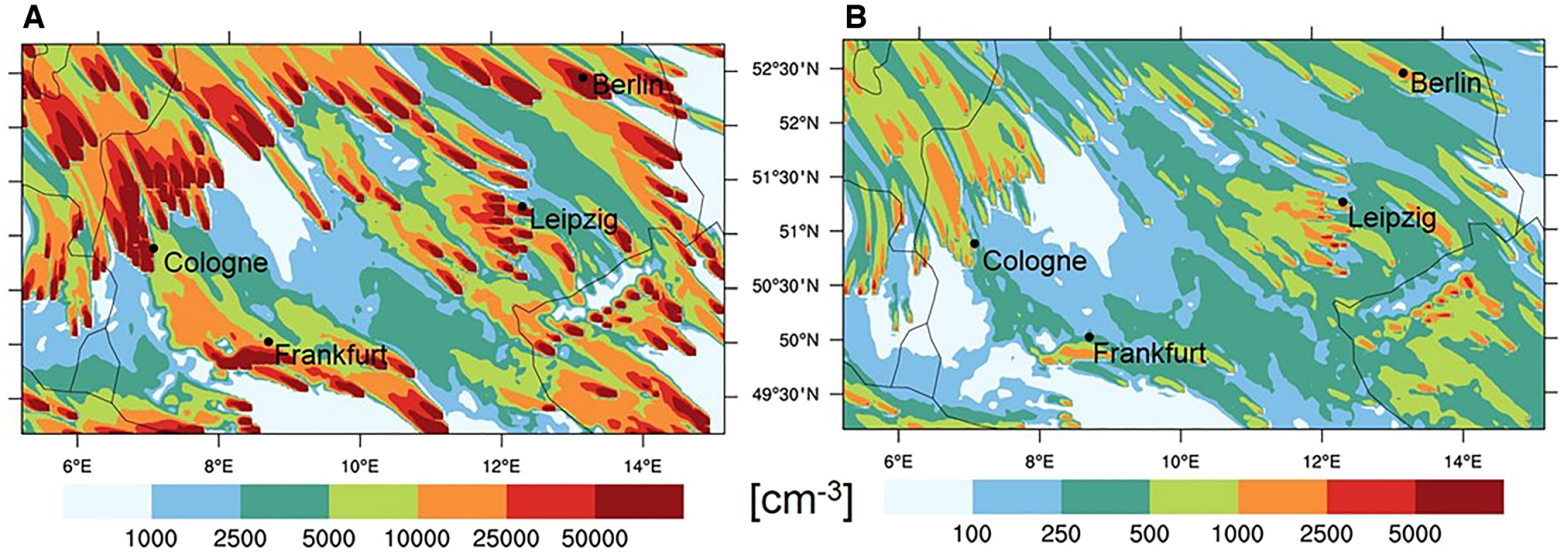
Comparison of primary Aitken mode particle plumes from power stations over northern Germany according to the German weather forecast model COSMO Art based on measured emissions (**A**) and on Y2000 (AeroCom, https://aerocom.met.no) based emissions (**B**) for southeasterly winds. The model was run for November conditions to suppress secondary gas to particle conversion (adapted from^14^).

The increased emissions of fossil fuel related ultrafine aerosols/CCN since ~ year 2000 have consequences for the regional climate up to several hundred km downwind of single sources. The elevated emission produces a patchy blanket of ultrafine particle plumes (see Fig. 4, adapted from^14^). From there, nano- and Aitken- particles and, a few hours later and under suitable fair weather conditions, CCN, are convectively mixed either up to cloud base or down to the ground^18,21^. Observations at the earth’s surface may thus give a glimpse on real particle number concentrations in the planetary boundary layer, but are likely not sufficient for regional or global budget estimates^10,18^.

The highly variable spatial UFP distribution agrees with the regional or continental variability of the hydrological cycle with regard to changes of rainfall intensity and timing, as well as river flows^4,20,25,62^. In a first step the smaller cloud droplets and the subsequent delay of raindrop production leads to a longer residence time of water vapour in the atmosphere. Any further processes in aerosol cloud interactions dependent on a variety of ambient variables and cloud types, normally not fully accessible to experimental investigations. Thus, it’s difficult to derive a clear causality for the final response of the hydrological cycle (Heinzeller 2016). But, it is interesting to note that most areas with strongly enhanced UFP emissions within our two decades of experiments show a declining overall rainfall trend coincidental to an increase of UFP.

Depending on size and updraft velocities in the clouds, the UFP resulting from flue gas cleaning technology (Figs. 1, 2) are likely too small for overall positive cloud modification effects under discussion for potential geoengineering and may even worsen global warming^56^. Global warming related to pure CO_2_ or other long-lived greenhouse gases already leads to more extreme weather patterns due to increased latent heat in the atmosphere^63^. The localized, but ubiquitous, fossil fuel related UFP emissions, their effect on the lifetime of water molecules in the atmosphere and their contribution to the water vapour (latent heat) abundance also in mid elevations of the atmosphere may play an additional role in explaining the increasingly fast warming observed within the last decade^64,65^, and exacerbate the crucial health conditions of the planet.

## Supplementary Information

Supplementary Information.

## Data Availability

Data sets used for budget studies and figures are available on 10.5281/zenodo.5137872.
