# Supplementary material for: Unprecedented levels of ultrafine particles, major sources, and the hydrological cycle
Source: Sci Rep. 2022 May 6;12:7410. doi: 10.1038/s41598-022-11500-5 (PMC9076833; doi:10.1038/s41598-022-11500-5)
Supplement: Supplementary file 1 — Supplementary Information. [file 41598_2022_11500_MOESM1_ESM.docx]

**Supplementary Information:**

**Unprecedented levels of ultrafine particles, major sources, and the hydrological cycle**

Wolfgang Junkermann^1*^, Jorg Hacker^2,3^

^1^Karlsruhe Institute of Technology, KIT, IMK-IFU, Garmisch-Partenkirchen, Germany

^2^Airborne Research Australia, Parafield Airport, South Australia

^3^College of Science and Engineering, Flinders University, Adelaide, South Australia

**Experimental setup**

For airborne surveys of fine, ultrafine and nanometer sized aerosols we used two different small well-instrumented environmental research aircraft (SERA). For observations all over Europe, Mexico and China a microlight aircraft ^S1,S2^ operated by the Karlsruhe Institute of Technology (KIT), Garmisch-Partenkirchen, Germany, was used. It’s aerosol package contains a scanning mobility particle spectrometer (SMPS) system for size distributions from 4.5 – 350 nm and an optical spectrometer for larger sizes 300 nm – 20 µm (GRIMM wide range aerosol spectrometer WRAS, 5403), an independent fast counter (1 s) for particles > 4.5 nm used for real-time flight guidance and quality check for the SMPS measurements, and a multi wavelength aethalometer (Magee AE42) for optical characterization, including black carbon (see Table S1). The reduction of visibility caused by fine particles, which is closely related to PM2.5 or PM10 is monitored by an AVMIII airborne visibility meter. A (micro)meteorology system ^S3^ as well as spectral and thermal radiation instrumentation ^S4^ are included. To support the meteorological analysis, surface and cloud base temperatures were recorded by downwards and upwards pointing infrared sensors. The aircraft is able to fly for ~ 6 h at a true air speed of ~90 km h^-1^ and up to 4 km altitude. Due to the larger distances in Australia a motorized glider (ECO-Dimona) operated by Airborne Research Australia (ARA) Parafield Airport, SA was used. This motorglider, having a range of > 1000 km is carrying the KIT aerosol package as detailed above in underwing pods. (Micro)meteorology is provided by a BAT (Best Aircraft Turbulence) probe^S5^.

**Estimation of particle source strength**

Emission of UFP was derived from excess number concentrations observed during plume cross sections times wind speed. Vertical homogeneity within the PBL and PBL thickness is derived from occasional vertical soundings from ~ 100 m AGL up to the free troposphere carried out before, during, and at the end of the flight patterns. The initial profile defines the PBL thickness and vertical planetary boundary structure, as well as homogenic mixing based on CPC counts, temperature and dewpoint and is used to decide about any subsequent horizontal flight levels (typically ~ 60% of the PBL thickness or flight patterns with shorter legs switching between above and below inversion level). A fast, 1 s, chilled mirror is used for dewpoint measurements.During horizontal transects, surface temperature serves as indicator for most likely convective initiation and intensity of vertical mixing; comparisons of potential temperature at flight level extrapolated to ground level and turbulent kinetic energy (TKE) serve asfurther indicators for convective mixing intensity. For the studies near Karlsruhe (ENBw) and near Kogan Creek, Australia, Lagrangian patterns were flown to investigate in plume particle growth or GPC contributions. In-situ wind measurements from the turbulence probe of the aircraft were compared during the postprocessing analysis to HYSPLIT backtrajectories as quality control for the wind measurement and a potential deviation of the transport pathway from the in-situ derived wind during the last 24 hours, important for source apportionment. For plume cross section and budget estimates, horizontal wind speeds between 3 and 10 m sec^-1^ proved to be a suitable range with high plume to background values.

**Table S1: Instrumentation package of the ultralight aircraft D-MIFU. The motorized glider used in Australia (VH-OBS or VH-EOS) carries a BAT Probe for micrometeorology and the D-MIFU aerosol package**

Parameter Instrument time resolution

Ozone UV-Photometer 10 sec

Act. flux 300 nm JO1D 2 filter-radiometers 1 sec

Act. flux 380 nm JNO2 2 filter-radiometers 1 sec

Global radiation (shortwave) 2 Pyranometers (LICOR, 210) 1 sec

Longwave (IR) radiation 2 Pyrgeometers, CGR4 1 sec

NDVI, 400, 550, 650, 990 nm 2 4 λ irradiance sensors (SKYE) 1 sec

Temperature Thermocouple 50 Hz

Dewpoint Chilled mirror (METEOLABOR) 1 sec

Relative humidity capacitive sensor SHT 75 30 sec

Pressure Capto SP28L sensor 20 Hz

Turbulence, 3D windvector noseboom-type probe 20 Hz

Ultrafine particles (#/cm-3) GRIMM CPC4 > 4.5 nm 1 sec

Fine particles / size 300 nm – 20 μm, GRIMM OPS 6 sec

Ultrafine particles / size SMPS 4.5-350 nm, GRIMM 2 min

Scatt. coeff. / visibility HSS-AVMIII (870 nm) 5 sec

Absorption coefficient 7 λ Aethalometer 370 – 950 nm 2 min

Attitude / Heading INS OXTS RT3102 < 0.05 ^o^, 100 Hz

Altitude above ground ULS < 600 m +- 2 cm 10 Hz

Surface temperature Infrared temperature sensor 1 sec

Sky temperature (> -50^o^C) Infrared temperature sensor 1 sec

For the CHARMEX Pre-campaign (VESSAER) over Corsica we installed an additional cloud condensation nuclei spectrometer provided by Greg Roberts,CNRM, UMR 3589, Météo-France/CNRS, Toulouse, France.


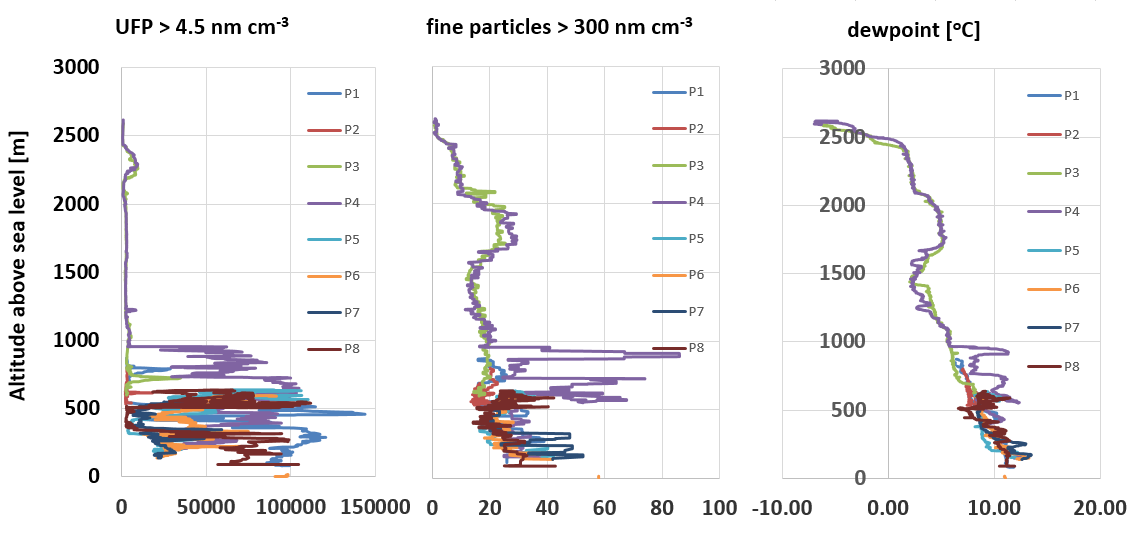


**Fig. S1**

Vertical profiles of UFP, fine particles (>300 nm) and dewpoint over the Islands of Malta and Gozo, with northerly winds (sector 360 – 30 degrees) downwind of the main shipping route on June 14, 2013. Profiles 3 and 4 were flown over the northeastern and southwestern part of Gozo, profile 8 during return to the airport. The international airport of Malta is located ~ 30 km downwind towards the southeast of Gozo.

**Table S2:**

**Aircraft campaigns with UFP, fine particle and SMPS measurements**

1998 – 2008 Italy, Po-valley, Lampedusa ^S1,S4,S5,S6^

2003 ESCOMPTE, Southern France ^S4^

2000 – 2004 QUEST, Ireland, Finland, Italy ^S8-S10^

2007 COPS, Germany ^S11^

2007-2009 MAGIM, Inner Mongolia ^S3^

2007-2011 BUFEX, Western Australia ^S12,S13^

2012 UPEC Australia, Queensland ^S14^

2012-2014 TUBA, Germany ^S15^

2014 BAERLIN, Germany ^S16^

2012/2013 VESSAER, CHARMEX, France ^S17^

Corsica, Malta

2017 BALTIC, Germany ^S17^

Campaign summary:

Italy, Po valley and Lampedusa, conditions covered heavy pollution air chemistry within the whole valley and new particle formation (NPF) events in the eastern part close to the Adriatic Sea. Lampedusa flights aimed at Saharan dust and radiation.

ESCOMPTE in southern France between Avignon, Marseille and Aix en Provence, focus on air chemistry and radiation with and without convective clouds (Junkermann 2005). Major UFP contribution from the Marseille-Beurre refineries.

QUEST aimed at new particle formation at Mace Head, Ireland, the boreal forest of Finland and the eastern Po Valley.

COPS was flown over the northern Black Forest, Germany, with the main objective to investigate thermal or orographic convection, relevant to precipitation and its drivers. UFP transport observed from the Rhine valley into the black forest along the valleys

MAGIM, Inner Mongolia, China was a campaign on CO_2_ and water vapour fluxes over the grasslands of Mongolia and led, coincidentially several times to the observation of power station plumes from Xilinhot ~70 km upwind. Inner Mongolia is subject to a pronounced rainfall decline (~ 4% per decade) since ~Y2000 in contrast to climate model results expecting an increase in rainfall by about 3% per decade.

BUFEX and UPEC in Australia were flown to compare two regions with similar rainfall changes (- 25% in 30 years) but totally different land use.

TUBA and BAERLIN were Germany-wide selected regional surveys to search for sources and their quantification. Germany suffers from drought and ground water depletion mainly in the east and northeast and exhibits an overall negative annual rainfall trend since 1990 (source GermanEnvironmental Agency).

Mediterranean marine aerosols were investigated several times with vertical profiles from sea level up to > 4500 m ASL over the Island of Lampedusa, close to the African coast for Saharan Dust investigations and over Corsica and Malta / Gozo within VESSAER/CHARMEX campaigns. Particle number concentrations in the central Mediterranean area between Malta and Sicily changed from ~ 800 – 1200 cm^-3^ in 1970 to ~ 25,000 cm^-3^ in 2013. Corsica is less affected by direct shipping emissions but still exhibits up to 8000 cm^-3^ depending on long-range transport. But, due to stronger thermal mixing over a large island planetary boundary layer may reach up to 3000 m AGL. Several countries in the Mediterranean suffer from long term summer droughts and a decline of river flows.

BALTIC was a direct investigation of the emissions from the ‘clean’ hybrid ferry with state of the art flue gas cleaning operating between Germany (Puttgarden) and Denmark (Rødby).

**Potential chemistry favouring internal new particle formation in power stations**

The suppression of NO_2_ in flue gas emission is often implemented via ammonia addition, either with selective catalytic reduction (SCR) or without an additional catalyst (SNCR) ^S18,S19^. Ammonia and NO_2_ or SO_2_ plus water vapour rapidly form particulate matter. Although a large fraction of this matter is removed by filtration, there is still enough mass left to form nucleation mode particles. The ammonia consumption of these processes can be calculated by stoichiometry, remaining NO_2_ emission and conversion efficiency ^S20^. For German power stations the industrial application of NH_3_ amounts to ~ 30% of the total annual emissions, yet these ammonia emissions in the solid phase as sulphates or nitrates are not appearing in any emission inventories.

References (Supplement)

S1. Junkermann, W., An ultralight aircraft as platform for research in the lower troposphere: system performance and first results from radiation transfer studies in stratiform aerosol layers and broken cloud conditions, *J. Ocean.& Atm. Techn*., **18**. 934-946, (2001)

S2. Junkermann, W., The actinic UV-radiation budget during the ESCOMPTE campaign 2001: Results of airborne measurements with the microlight research aircraft D-MIFU, *Atmospheric Research*, **74**, 461 – 475, (2005), doi:10.1016/j.atmosres.2004.06.009

S3. Metzger, S., et al. Spatial resolution and regionalization of airborne flux measurements using environmental response functions, *Biogeosciences*, **10**, 2193-2217, (2013), doi:10.5194/bg-10-2193-2013

S4. Meloni, D., Tropospheric aerosols in the Mediterranean: II Radiative effects through model simulations and measurements; *Journal of Geophysical Research,* **108**(D10), 4317, doi:10.1029/2002JD002807, (2003)

S5. Hacker, J. M., and Crawford T. L., The BAT-probe: The ultimate tool to measure turbulence from any kind of aircraft (or sailplane). *Tech. Soar*., **23**, 42–45 (1999)

S6. Di Iorio, T. et al., Tropospheric aerosols in the Mediterranean: I. Microphysical and optical properties, *Journal of Geophysical Research*, **108**(D10), 4316, doi:10.1029/2002JD002815, (2003)

S7. di Sarra, A., et al., Radiation, ozone, and aerosol measurements at Lampedusa During the PAUR II campaign, in IRS 2000: Current Problems in Atmospheric Radiation, W. L. Smith and Yu. M. Timofeyev (Eds.). A. Deepak Publishing, Hampton, Virginia. 1193-1196, (2002)

S8. Laaksonen, A. *et al.*, Cloud Condensation Nucleus Production from Nucleation Events at a Highly Polluted Region, *Geophys. Res. Lett*., **32**, L06812, 10.1029/2004GL022092, (2005)

S9. O'Dowd, C. D., et al. Airborne measurements of nucleation mode particles I: coastal nucleation and growth rates, *Atmos. Chem. Phys*., **7**, 1491-1501, (2007)

S10. O'Dowd, C. D., et al. Airborne measurements of nucleation mode particles II: boreal forest nucleation events, *Atmos. Chem. Phys*., **9**, 937–944, https://doi.org/10.5194/acp-9-937-2009, (2009)

S11. Junkermann, W., Hagemann, R., and Vogel, B., Nucleation in the Karlsruhe plume during the COPS / TRACKS - Lagrange experiment, *QJRMS,* **137**, 267-274, (2011)

S12. Junkermann W., Hacker J., Lyons T., and Nair U., Land use change suppresses precipitation, *Atmos. Chem. Phys*., **9**, 6531-6539, (2009) [www.atmos-chem-phys.net/9/6531/2009/](http://www.atmos-chem-phys.net/9/6531/2009/)

S13. Heinzeller, D., Junkermann, W. and Kunstmann, H., Anthropogenic aerosol emissions and rainfall decline in South-West Australia: coincidence or causality, *Journal of Climate*, **29**, 8471-8493, (2016), DOI: 10.1175/JCLI-D-16-0082.1

S14. Junkermann, W., and Hacker, J.M., Ultrafine particles over Eastern Australia: an airborne survey, *Tellus B*, **67**, 25308, (2015)

http://dx.doi.org/10.3402/tellusb.v67.25308

S15. Junkermann, W., Vogel, B. and Bangert, M., 2016, Ultrafine particles over Germany - an aerial survey, *Tellus B*, **68**, 29250, (2016)

http://dx.doi.org/10.3402/tellusb.v68.29250

S16. Bonn, B., et al. BAERLIN2014 – the influence of land surface types on and the horizontal heterogeneity of air pollutant levels in Berlin, *Atmos. Chem. Phys*., **16**, 7785-7811, (2016) doi:10.5194/acp-16-7785-2016

S17. Junkermann, W.: Ultrafine particle emissions in the Mediterranean, In: Atmospheric Chemistry in the Mediterranean – Vol. 2, From Air Pollutant Sources to Impacts, Dulac, F., Sauvage, S., and Hamonou, E., Eds., Springer, in press, 2021

S18. Bai, H., Biswas, P., and Keener, T.C. Particle Formation by NH_3_-S0_2_ Reactions at Trace Water Conditions, *Ind. Eng. Chem. Res*. **31**, 88-94 (1992)

S19. Srivastava, R.K., Hall, R.E., Khan S., Culligan, K., and Lanl, B.W., Nitrogen Oxides Emission Control Options for Coal-Fired Electric Utility Boilers, *J. Air & Waste Manage*. *Assoc.* **55**:1367–1388 (2005)

S20. Shanghai Electric, 2019, https://www.shanghai-electric.com/group_en/upload/resources/file/2019/11/07//78654.pdf, last visit April (2021)
